# Burden of HIV and treatment outcomes among TB patients in rural Kenya: a 9-year longitudinal study

**DOI:** 10.1186/s12879-023-08347-0

**Published:** 2023-05-30

**Authors:** Moses M. Ngari, Mohammed A. Rashid, Deche Sanga, Hiram Mathenge, Oscar Agoro, Jane K. Mberia, Geoffrey G. Katana, Michel Vaillant, Osman A. Abdullahi

**Affiliations:** 1 KEMRI/Wellcome Trust Research Programme, P.O Box 230, Kilifi, 80108 Kenya; 2 Department of Public Health, Pwani University, Kilifi, Kenya; 3 Ifakara Health Institute, Bagamoyo, Tanzania; 4 Kilifi County TB Control Program, Kilifi, Kenya; 5 Nyeri County TB Control Program, Nyeri, Kenya; 6 School of Health Sciences, Meru University of Sciences and Technology, Meru, Kenya; 7 Kilifi County Department of Public Health, Kilifi, Kenya; 8 Competence Center for Methodology and Statistics, Luxembourg Institute of Health, Luxembourg, Luxembourg

**Keywords:** Tuberculosis, HIV, Treatment outcomes

## Abstract

**Background:**

Although tuberculosis (TB) patients coinfected with HIV are at risk of poor treatment outcomes, there is paucity of data on changing trends of TB/HIV co-infection and their treatment outcomes. This study aims to estimate the burden of TB/HIV co-infection over time, describe the treatment available to TB/HIV patients and estimate the effect of TB/HIV co-infection on TB treatment outcomes.

**Methods:**

This was a retrospective data analyses from TB surveillance in two counties in Kenya (Nyeri and Kilifi): 2012‒2020. All TB patients aged ≥ 18 years were included. The main exposure was HIV status categorised as infected, negative or unknown status. World Health Organization TB treatment outcomes were explored; cured, treatment complete, failed treatment, defaulted/lost-to-follow-up, died and transferred out. Time at risk was from date of starting TB treatment to six months later/date of the event and Cox proportion with shared frailties models were used to estimate effects of TB/HIV co-infection on TB treatment outcomes.

**Results:**

The study includes 27,285 patients, median (IQR) 37 (29‒49) years old and 64% male. 23,986 (88%) were new TB cases and 91% were started on 2RHZE/4RH anti-TB regimen. Overall, 7879 (29%, 95% 28‒30%) were HIV infected. The proportion of HIV infected patient was 32% in 2012 and declined to 24% in 2020 (trend *P*-value = 0.01). Uptake of ARTs (95%) and cotrimoxazole prophylaxis (99%) was high. Overall, 84% patients completed six months TB treatment, 2084 (7.6%) died, 4.3% LTFU, 0.9% treatment failure and 2.8% transferred out. HIV status was associated with lower odds of completing TB treatment: infected Vs negative (aOR 0.56 (95%CI 0.52‒0.61) and unknown vs negative (aOR 0.57 (95%CI 0.44‒0.73). Both HIV infected and unknown status were associated with higher hazard of death: (aHR 2.40 (95%CI 2.18‒2.63) and 1.93 (95%CI 1.44‒2.56)) respectively and defaulting treatment/LTFU: aHR 1.16 (95%CI 1.01‒1.32) and 1.55 (95%CI 1.02‒2.35)) respectively. HIV status had no effect on hazard of transferring out and treatment failure.

**Conclusion:**

The overall burden of TB/HIV coinfection was within previous pooled estimate. Our findings support the need for systematic HIV testing as those with unknown status had similar TB treatment outcomes as the HIV infected.

**Supplementary Information:**

The online version contains supplementary material available at 10.1186/s12879-023-08347-0.

## Background

Despite the ambitious global targets to reduce TB deaths and incidence rate by 95% and 90% respectively from 2015 to 2035, TB still remains a major global public health problem [1, 2]. The reported number of new TB cases dropped by 18% from 7.1million in 2019 to 5.8million in 2020 while TB deaths increased by approximately 8% (from 1.2 in 2019 to 1.3million in 2020) among HIV-negative and by 2.4% among HIV-positive patients (from 209,000 in 2019 to 214,000 in 2020) [3]. This has been attributed to the disruption in health services caused by the COVID-19 pandemic [3]. In 2021, the reported number of TB cases have increased to 6.4 million, numbers still lower than those in 2019 [4]. HIV has been identified as significant risk factor estimated to contribute ~ 17% of TB burden in Kenya [5].

To end TB, the focus now shifts to `high risk’ group like the TB patients coinfected with HIV [6]. Among people living with HIV (PLHIV), TB is one of the top killers, with at least one in four deaths among PLHIV being attributable to TB [6, 7]. In 2010, World Health Organization (WHO) published priority research questions for this group (TB/HIV) and a policy document on collaborative TB/HIV management in 2012 [8]. Among other recommendations, the policy recommended routine HIV testing among all patients diagnosed or with presumptive TB but the recommendation was based on low quality evidence [8]. Kenya is among 30 high burden TB countries with high prevalence of TB/HIV [5].

In 2013, a systematic review and meta-analysis of prevalence of TB/HIV co-infection reported a pooled estimate of 24% which varied across continents [9]. The pooled prevalence of TB/HIV co-infection was highest in Africa (31%) and lowest in USA (15%). However, the systematic review excluded studies from China and did not report TB treatment outcomes [9]. In another systematic review and meta-analysis in Sub-Saharan Africa only in 2019, the pooled estimate of TB/HIV co-infection was 32% but varied across the Africa regions (was 44% in Southern Africa, 41% in Central Africa, 31% in Eastern and 26% in Western Africa) [10]. In Ethiopia, the pooled prevalence of TB/HIV from different regions within the country was 23% in 2019 [11]. Despite recent increased uptake of cotrimoxazole preventive therapy (CPT) and antiretroviral therapy (ART), mortality rates among TB/HIV patients has remained high, for example it was 16% in Swaziland in 2016 (uptake of CPT was 99% and 75% for ART) [12]. In Kenya, the mortality among TB/HIV on ART was 10.3% (ART uptake was 87%) in 2016 [13] while in Kilifi county, Kenya it was 9.9% among TB/HIV on ART and 17% among those not on ART (CPT uptake was 94% and ART was 99%) in 2019 [14].

Most of these previous studies have not reported TB treatment outcomes disaggregated by HIV status. It is likely the TB/HIV prevalence could also have changed over time because of the vast investment in controlling and preventing HIV or the increased access to ART by PLHIV [15, 16]. Over the years, policy of HIV testing in Kenya has changed from self-initiated voluntary counselling & testing (VCT) to provider-initiated testing & counselling (PITC) [17, 18]. Since 2016, Kenya changed the policy of starting ART to immediate initiation after HIV diagnosis regardless of CD4 counts based on the WHO recommendation and emerging scientific evidence showing benefit of `test and treat’ approach [19–21]. Isoniazid preventive therapy (IPT) for PLHIV to protect against TB [22] was recommended by WHO and implemented in Kenya from 2015 [23]. There is paucity of data on changing trends of TB/HIV prevalence and their TB treatment outcomes. This study aims to: a) estimate the burden of TB/HIV co-infection over time, b) describe the treatment available to TB/HIV patients and c) estimate the effect of TB/HIV co-infection on TB treatment outcomes using TB surveillance data from two counties in Kenya (Nyeri and Kilifi) from 2012 to 2020.

## Methods

### Study design

This study was a retrospective secondary data analyses of routine standard National Leprosy and Tuberculosis and Lung Disease (NTLD) register data.

### Study settings

Routine Data collected through the TB electronic surveillance system from all sub-counties in the Kilifi and Nyeri counties were used. The Kilifi County is located along the Kenya coast and had a population of approximately 1.4million people in the 2019 census. The population density in Kilifi in 2019 was 116 people per Sq.Km. The major economic activities in Kilifi County are tourism and fishing because of the proximity to Indian Ocean. The county is served by one County referral hospital and a total of 235 health facilities. The HIV prevalence in Kilfi County was 4% in 2020 [24].

Nyeri county is located in central Kenya and had a population of 750,000 people in 2019 census. In 2019, the county population density was 228 people per Sq.Km. Agriculture is the main economic activities in the county, with coffee and tea farming being the top cash crops. The county is served by one County Referral Hospital and a total of 190 health facilities. Nyeri had HIV prevalence of 3.7% in 2018 [25].

### TB diagnosis and treatment

Both counties treat suspected/confirmed TB cases following the Kenya national guidelines [26]. TB is diagnosed bacteriologically using smear microscopy, culture or WHO-recommended molecular TB diagnostics (Xpert MTB/RIF) or clinically by a medical practitioner based on clinical symptoms, X-ray abnormalities, suggestive histology or extrapulmonary cases without laboratory confirmation. New TB patients who have not been on anti-TB previously, are started on Rifampicin (R), Isoniazid (H), Pyrazinamide (Z) and Ethambutol (E) for two months followed by Rifampicin (R) and Isoniazid (H) for four months (2RHZE/4RH). New patients diagnosed with TB meningitis and osteo-articular TB are treated as above except that they take Rifampicin (R) and Isoniazid (H) for ten months (2RHZE/10RH). Retreatment patients who had previously been on anti-TB for more than one month including those who relapsed are started on Streptomycin (S), Ethambutol (E), Rifampicin (R), Isoniazid (H) and Pyrazinamide (Z) for 2 months, followed by Ethambutol (E), Rifampicin (R), Isoniazid (H) and Pyrazinamide (Z) for one month and five months of Ethambutol (E), Rifampicin (R) and Isoniazid (H) (2SRHZE/1RHZE/5RHE). Treatment of drug resistant TB depends on the pattern of drug resistance and follows the WHO 2018 and Kenya National guidelines [27, 28]. Following the WHO 2018 guidelines, Kenya transitioned to injectable free regimens for drug resistant TB in January 2020 [28]. The treatment uses standardized regimen which can be individualized subject to patient response. For example, multidrug-resistant/rifampicin-resistant (MDR/RR TB) would receive 5 drugs (Bedaquiline, Clofazimine, Levofloxacin, Cycloserine and Linezolid) for six months intensive phase and three drugs (Clofazimine, Levofloxacin and Cycloserine) for 12 months continuation phase [28]. All TB patients starting treatment are systematically offered HIV testing and counselling as standard of care. HIV testing is conducted by the Comprehensive Care Center (CCC) which offers a package of HIV management care: counselling and testing for HIV, diagnose, treatment and management of opportunistic infections, treatment adherence counselling, nutrition counselling and delivery of ARTs. Rapid antibody tests are offered to all TB patients at the CCC using the routine provider-initiated HIV testing and counselling (PITC) approach. Those who test positive for HIV are put on cotrimoxazole preventive therapy (CPT) and ART irrespective of CD4 count. In cases, where there is HIV rapid diagnosis test (RDT) kit stock out or patient decline consent, TB patients may miss being tested or get tested during TB treatment. Both TB and HIV diagnosis and treatment are readily available and offered for free in all government health facilities in Kenya.

### Study population

The study population was all adult TB patients (≥ 18 years) who were on anti-TB treatment from January 2012 to December 2020 within the Kilifi and Nyeri counties. The two counties were selected because they characterise two different environmental aspects of Kenya, their data were accessible, and they represent varying TB incidence and economic status [13].

### Data source and variables

Data were extracted from the TB Electronic surveillance system known as Treatment Information from Basic Unit (TIBU) from the two counties. TB treatment outcomes follow the World Health Organization (WHO) classification: cured, treatment complete, failed treatment (i.e., remaining smear-positive after 5 months of treatment), defaulted/lost-to-follow-up (LTFU), died and transferred out [29]. In this routine surveillance data, both cured and those who completed six months of treatment were categorised as treatment complete. The main exposure was HIV comorbidity. Other exposures examined included demographic (age, sex), county of residence, year of starting TB treatment, nutritional status (body mass index), nutritional support provided and clinical features (other underlying comorbidities, type of TB (pulmonary or extra-pulmonary), TB diagnosis (bacteriological confirmed TB or empirically treated), treatment regimen and direct observed treatment.

### Study size

The study used all available eligible patient data from 2012 to 2020. Data from 27,285 suspected TB patients, 15,974 from Kilifi County and 11,311 from Nyeri County were included in the analyses. The study size (*N* = 27,285) was adequate to show a proportion of 30% with HIV co-infection, assuming precision level of 5% giving a 95% confidence interval (25% to 35%) and a two-tailed alpha of 0.05 [14, 30].

### Statistical analysis

Extracted data were cleaned, curated and merged into one dataset. The pattern of any missing data were assessed and missingness assumed not to be at random. We created categorical variables for variables with missing values and added extra category for missing/unknown, for example HIV status and Body Mass Index (BMI) have unknown categories. We used data up to December 2020 to ensure all the patients had completed their treatment and their treatment outcome available at the time of analysis. Continuous variables were categorized into WHO standard categories, for example BMI was categorised into: undernourished (BMI < 18.5), normal (BMI 18.5 to 25) and overweight (BMI ≥ 25).

The burden of TB/HIV in patients aged > 18 years of the two counties was reported as proportion with binomial exact 95% confidence interval. We used a non-parametric Wilcoxon-type test for trend to examine if there has been a declining/increasing trend in proportion of TB/HIV among patients on TB treatment from 2012 to 2020 [31]. To test the effect of TB/HIV coinfection on TB treatment completion (both cured or completed at least six months treatment), multilevel mixed-effects logit regression with the county variable as random intercept was used and Odds Ratios (OR) reported as measure of effect. To examine the effect of TB/HIV coinfection on death, defaulting treatment/Lost-to-follow-up (LTFU), transfer out of the two counties and treatment failure, survival analysis models were used. Time at risk was from date of starting TB treatment up to six months later (for those who completed treatment) or date of the event. The proportional-hazards (PH) assumption was tested using scaled Schoenfeld residuals for each outcome separately. There was no evidence of PH violation for all the outcomes (Supplementary Table 1). To control for the unobserved heterogeneity between the two counties, we used Cox Proportional regression model with shared frailty (county) assuming the frailties had a gamma distribution. In all the regression models, we conducted univariate analyses by only including HIV status as the independent variable. In the multivariable regression models, we adjusted for other collected exposure variables as a priori confounders. Separate regression models were conducted for each TB treatment outcome. Time to event were plotted using Kaplan–Meier curves.

Sub-group analyses evaluating the effect of HIV confection on TB treatment outcomes including only HIV infected patients on both ARTs and CPT were conducted by using a Cox Proportional Hazard model. As for sensitivity analyses, we classified WHO TB treatment outcomes into a binary variable: a) successful outcome (cured/completed six months under treatment) and b) poor outcome (combined deaths, defaulting/LTFU, treatment failure and transfer out) and examined the effect of HIV status in a logistic model. In another sensitivity analysis, we tested effect modification of HIV status on the association between various other exposure variables and TB treatment outcomes by comparing regression models with and without interaction term using likelihood ratio test. All statistical analyses were conducted using STATA version 17.0 (StataCorp, College Station, TX, USA).

### Ethical consideration

Ethical approval to conduct the study was granted by the Pwani University Ethics Review Committee (ERC/PU-STAFF/003/2022). The analyses used anonymised data. Study participants provided written informed consent for their data to be used. The study was conducted following STrengthening the Reporting of OBservation studies in Epidemiology (STROBE) [32] and REporting of studies COnducted using Observational Routinely-collected health Data (RECORD) [33].

## Results

In the two counties, there were 31,470 TB patients started on treatment from 2012 to 2020. We excluded 4185 (13%) because they were aged < 18 years at the time of starting TB treatment and therefore included 27,285 TB patients (15,974 (59%) from Kilifi and 11,311 (41%) from Nyeri County).

The majority patients were male (*N* = 17,450, 64%) and from the main economically active age group of 18 to 50 years (*N* = 21,340, 78%). New TB cases (*N* = 23,986, 88%) were the most frequent patient type. There were 23,639 (87%) pulmonary TB cases and 2434 (13%) had extrapulmonary TB. Directly observed therapy (DOT) by the family members (*N* = 24,842, 91%) was the most frequent treatment strategy. Slightly more than one half (*N* = 14,082, 52%) had normal BMI (BMI of 18 to 24.9). Fourteen thousand and sixty-seven (52%) had a bacteriologically confirmed diagnosis of TB while 13,218 (48%) were diagnosed using WHO clinical signs or using abnormal Xray suggestive of TB. In line with the Kenya national guidelines majority of TB patients who were new patients were started on 2RHZE/4RH anti-TB regimen (*N* = 24,898, 91%) (Table 1).

**Table 1:** Study participants characteristics at the time of starting TB treatment

**Features**	**HIV negative (*N* = 19,019)**	**HIV positive (*N* = 7879)**	**HIV status unknown (N = 387)**	**All patients (*N* = 27,285)**
Sex				
Male	13,253 (70)	3930 (50)	267 (69)	17,450 (64)
Female	5766 (30)	3949 (50)	120 (31)	9835 (36)
Age in years				
18 to 30 years	6853 (36)	1700 (22)	121 (31)	8674 (32)
31 to 40 years	4629 (24)	2974 (38)	93 (24)	7696 (28)
41 to 50 years	2780 (15)	2125 (27)	65 (17)	4970 (18)
51 + years	4757 (25)	1080 (14)	108 (28)	5945 (22)
Patient type				
New cases	16,801 (88)	6821 (87)	364 (94)	23,986 (88)
Re-treatment cases	2218 (12)	1058 (13)	23 (5.9)	3299 (12)
TB type				
Pulmonary	16,585 (87)	6732 (85)	322 (83)	23,639 (87)
Extrapulmonary	2434 (13)	1147 (15)	65 (17)	3646 (13)
Recruitment health facility				
Public	15,429 (81)	5691 (72)	295 (76)	21,415 (78)
Private	3183 (17)	2070 (26)	87 (22)	5340 (20)
Prisons	407 (2.1)	118 (1.5)	5 (1.3)	530 (1.9)
DOT				
Family-based	17,369 (91)	7098 (90)	375 (97)	24,842 (91)
Community volunteer	645 (3.4)	246 (3.1)	2 (0.5)	893 (3.3)
Health worker	1005 (5.3)	535 (6.8)	10 (2.6)	1550 (5.7)
Nutrition status				
Undernourished	6080 (32)	2290 (29)	98 (25)	8468 (31)
Normal BMI	9738 (51)	4170 (53)	174 (45)	14,082 (52)
Overweight	2151 (11)	932 (12)	37 (9.6)	3120 (11)
Not reported	1050 (5.5)	487 (6.2)	78 (20)	1615 (5.9)
Method of TB diagnosis				
Clinical signs	8356 (44)	4658 (59)	204 (53)	13,218 (48)
Bacteriological confirmed	10,663 (56)	3221 (41)	183 (47)	14,067 (52)
Treatment regimen				
2RHZE/4RH	17,674 (93)	6875 (87)	349 (90)	24,898 (91)
2SRHZE/1RHZE/5RHE	971 (5.1)	832 (11)	10 (2.6)	1813 (6.6)
2RHZ/4RH	296 (1.6)	95 (1.2)	24 (6.2)	415 (1.5)
Others	78 (0.4)	77 (1.0)	4 (1.0)	159 (0.6)
County				
Kilifi	11,256 (59)	4553 (58)	165 (43)	15,974 (59)
Nyeri	7763 (41)	3326 (42)	222 (57)	11,311 (41)
Year of diagnosis				
2012	2408 (13)	1174 (15)	67 (17)	3649 (13)
2013	2165 (11)	1032 (13)	52 (13)	3249 (12)
2014	2132 (11)	1085 (14)	78 (20)	3295 (12)
2015	2061 (11)	872 (11)	21 (5.4)	2954 (11)
2016	1677 (8.8)	755 (9.6)	25 (6.5)	2457 (9.0)
2017	2004 (11)	774 (9.8)	27 (7.0)	2805 (10)
2018	2539 (13)	922 (12)	27 (7.0)	3488 (13)
2019	2284 (12)	711 (9.0)	42 (11)	3037 (11)
2020	1749 (9.2)	554 (7.0)	48 (12)	2351 (8.6)

*DOT* Direct observed treatment, *BMI* Body Mass Index, *ARVs* Antiretroviral, new patients have never been treated for TB or taken anti-TB drugs for less than one month, Re-treatment cases have received one month or more of anti-TB drugs in the past and includes relapsed patients, all results are N(%)

### TB/HIV coinfection

Overall, 7879/27285 (29%, 95%CI 28 − 30%) were HIV positive; 4553/15974 (29%, 95%CI 28 − 30%) from Kilifi county and 3326/11311 (29%, 95%CI 28 − 30%) from Nyeri county. A total of 387 (1.4%, 95%CI 1.3‒1.6%) had unknown HIV status. Table 1 shows patients characteristics stratified by HIV status.

### TB/HIV coinfection trend over the years

**Fig. 1:**
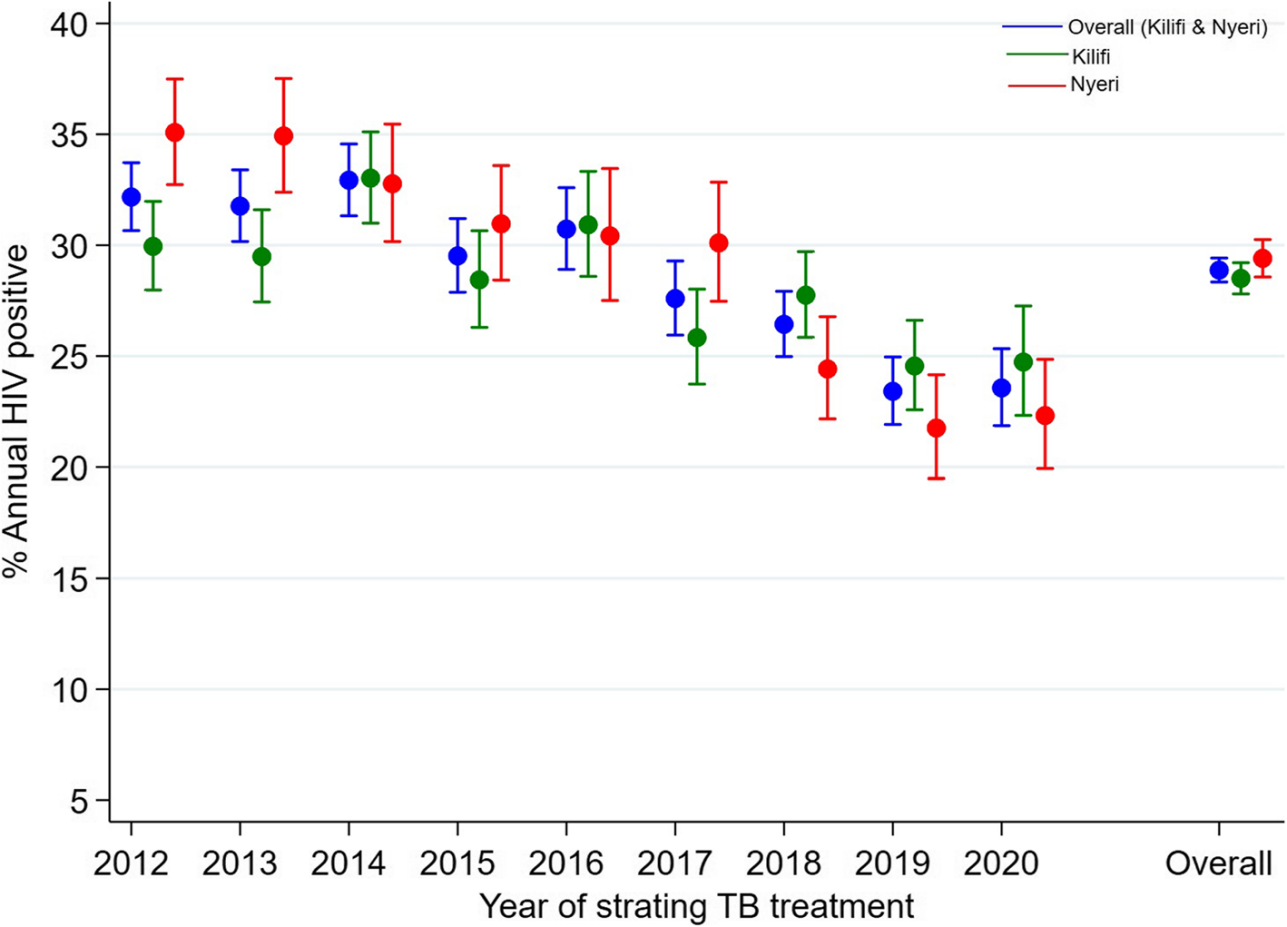
Annual proportion of HIV infected patients The scatter plot represents the point proportion and their 95% confidence intervals, test for trend *P*-vales are 0.01 overall, 0.03 for Kilifi county and 0.005 for Nyeri county

Overall, the proportion of HIV positive patient was 32% in 2012, plateauing between 2012 and 2014, thereafter declining to a low of 24% in 2020 (trend *P*-value = 0.01). In Kilifi county, the proportional of HIV positive was 30% in 2012 and declined to 25% in 2020 (trend *P*-value = 0.03) while in Nyeri county it declined from 35% in 2012 to 22% in 2020 (trend *P*-value = 0.005) Fig. 1.

Overall, 7449/7879 (95%) HIV positive patient were on ARTs and 7790/7879 (99%) were on cotrimoxazole prophylaxis. The high uptake of both ARTs and cotrimoxazole prophylaxis was similar between the two counties. Approximately one quarter (*N* = 1985, 25%) of all HIV infected patients were diagnosed within two weeks before and after starting TB treatment with 876 (11%) being diagnosed on the day of starting TB treatment (Table 2).

**Table 2:** Diagnosis of TB at the time of starting anti-TB treatment

	**County**		**All HIV infected (*N* = 7879)**
	**Kilifi (*N* = 4553)**	**Nyeri (*N* = 3326)**	
**On ARTs − N (%)**			
No	215 (4.7)	215 (6.5)	430 (5.5)
Yes	4338 (95)	311 (94)	7449 (95)
**On Cotrimoxazole prophylaxis − N (%)**			
No	33 (0.7)	56 (1.7)	89 (1.1)
Yes	4520 (99)	3270 (98)	7790 (99)
**Time between HIV diagnosis and starting TB treatment**			
> year before starting treatment	676 (15)	518 (16)	1194 (15)
Within 1 year and 15 days before starting treatment	543 (12)	434 (13)	977 (12)
Within two weeks before treatment	503 (11)	372 (11)	875 (11)
Same day of starting TB treatment	475 (10)	401 (12)	876 (11)
Within two weeks after starting treatment	99 (2.2)	135 (4.1)	234 (3.0)
> 2 weeks but before six months after starting treatment	77 (1.7)	106 (3.2)	183 (2.3)
Dates of HIV diagnosis not reported	2180 (48)	1360 (41)	3540 (45)

All results are N (%)

### TB Treatment outcomes

Overall, 22,994 (84%) patients completed six months of TB treatment as planned; 16,412 (86%) among the HIV negative, 6281 (80%) among HIV infected and 301 (78%) among those with unknown HIV status. There were 2084 (7.6%) deaths in the study, 1055 (5.6%) among HIV negative patients, 981 (12%) among HIV infected and 48 (12%) among those with unknown HIV status. A total of 1179 (4.3%) patients were lost-to-follow-up or defaulted treatment, 828 (4.4%) among HIV negative vs 328 (4.2%) among HIV infected. Other TB treatment outcomes stratified by HIV status are shown on Table 3.

**Table 3:** TB treatment outcomes after six months of anti-TB treatment stratified by HIV status

**TB treatment outcome**	**HIV negative (*N* = 19,019)**	**HIV positive (*N* = 7879)**	**HIV status unknown (*N* = 387)**	**All patients (*N* = 27,285)**
Treatment complete	16,412 (86)	6281 (80)	301 (78)	22,994 (84)
Treatment failure	187 (1.0)	62 (0.8)	4 (1.0)	253 (0.9)
Died	1055 (5.6)	981 (12)	48 (12)	2084 (7.6)
Defaulted/Lost-to-follow-up	828 (4.4)	328 (4.2)	23 (5.9)	1179 (4.3)
Transfer out	537 (2.8)	227 (2.9)	11 (2.8)	775 (2.8)

Treatment complete are patients who completed six months of treatment and those declared as cured, all results are N (%)

### Effect of HIV on TB treatment outcomes

HIV infection was associated with lower odds of completing TB treatment; HIV positive Vs negative (crude odds ratio (COR) 0.62 (95%CI 0.58‒0.67) and HIV unknown vs negative (COR 0.57 (95%CI 0.44‒0.71). In the multivariable model adjusted for confounders listed in Table 4, both HIV positive and unknown status were significantly associated with lower odds of completing TB treatment; HIV positive Vs negative (adjusted odds ratio (aOR) 0.56 (95%CI 0.52‒0.61) and HIV unknown vs negative (aOR 0.57 (95%CI 0.44‒0.73). Full multivariable model results are shown in Supplementary Table 2.

**Table 4:** Univariate and multivariable analysis of the effect of HIV status on TB treatment outcomes

**TB treatment outcome**	**Univariate analysis**		**Multivariable analysis**	
	**Crude Odds Ratio (95% CI)**	***P*-value**	**Adjusted Odds ratio (95% CI)**^**a**^	***P*-value**
**Treatment complete**				
HIV positive Vs negative	0.62 (0.58‒0.67)	< 0.001	0.56 (0.52‒0.61)	< 0.001
HIV unknown Vs negative	0.56 (0.44‒0.71)	< 0.001	0.57 (0.44‒0.73)	< 0.001
	**Crude HR (95% CI)**		**Adjusted HR (95% CI)**^**a**^	
**Died**				
HIV positive Vs negative	2.32 (2.13‒2.53)	< 0.001	2.40 (2.18‒2.63)	< 0.001
HIV unknown Vs negative	2.34 (1.75‒3.12)	< 0.001	1.93 (1.44‒2.56)	< 0.001
**Defaulted/Lost-to-follow-up**				
HIV positive Vs negative	1.01 (0.88‒1.14)	0.97	1.16 (1.01‒1.32)	0.04
HIV unknown Vs negative	1.58 (1.04‒2.39)	0.03	1.55 (1.02‒2.35)	0.04
**Transfer out**				
HIV positive Vs negative	1.06 (0.91‒1.24)	0.48	1.09 (0.92‒1.29)	0.30
HIV unknown Vs negative	1.08 (0.59‒1.96)	0.80	1.01 (0.56‒1.84)	0.97
**Treatment failure**				
HIV positive Vs negative	0.85 (0.64‒1.13)	0.27	1.08 (0.80‒1.46)	0.62
HIV unknown Vs negative	1.19 (0.44‒3.20)	0.73	1.54 (0.57‒4.15)	0.40

^a^Adjusted for a priori confounders: age, sex, patient type, TB type, facility type, method of DOT, BMI, TB treatment regimen, type of TB diagnosis, presence of other underlying medical conditions and year of diagnosis, Odds Ratios are from multilevel logit regression model, Hazard Ratios are from Cox proportional hazard regression models

**Fig. 2:**
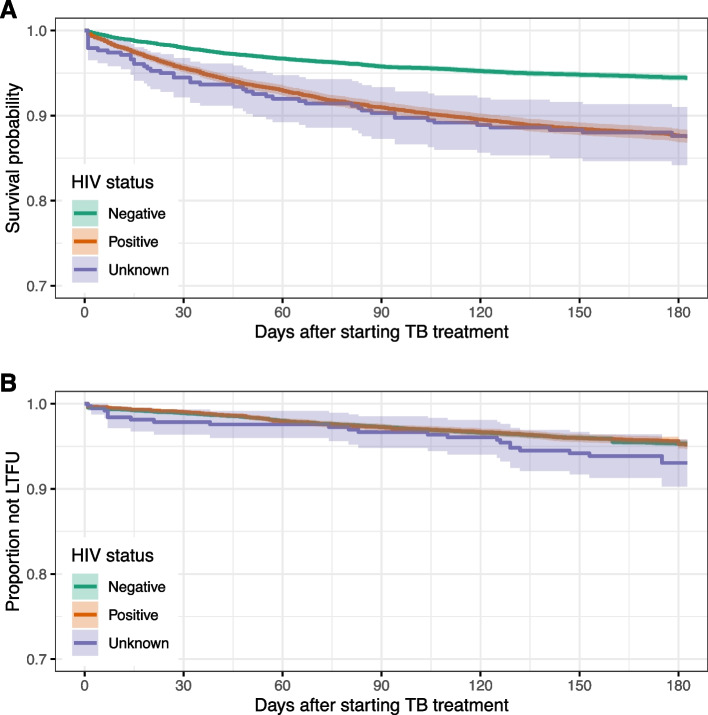
Kaplan Meier curves of: **a**) deaths and **b**) default/lost-to-follow-up stratified by HIV status

The patients were on follow-up for 11,965 person-years during the six months of treatment of which 2084 deaths occurred, mortality rate of 174 (95%CI 167‒182) deaths/1000 person-years. The mortality rates for HIV negative, positive and unknown status were 125 (95%CI 117‒132), 294 (95%CI 276‒313) and 304 (95%CI 229‒403) deaths/1000 person-years respectively. Both HIV positive and unknown were associated with higher hazard of death: HIV positive vs negative (adjusted Hazard ratio (aHR) 2.40 (95%CI 2.18‒2.63) and HIV unknown vs negative (aHR 1.93 (95%CI 1.44‒2.56)) Fig. 2a. The hazard of defaulting treatment/LTFU was higher among HIV infected or those with unknown status: HIV positive vs negative (aHR 1.16 (95%CI 1.01‒1.32) and HIV unknown vs negative (aHR 1.55 (95%CI 1.02‒2.35)) Fig. 2b. HIV status had no effect on hazard of transferring out and treatment failure (Table 4). Full multivariable models’ results are shown in Supplementary Tables 3 and 4.

In a sub-analysis including only HIV infected patients who had started ARTs and CPT, there was no significant change in the direction and effect of HIV on TB treatment outcomes (Supplementary Table 5). In the sensitivity analyses where the TB treatment outcomes was a binary variable (successful or poor), HIV status was positively associated with poor TB treatment outcome in a multivariable model adjusted for all a priori confounders listed in Table 4: HIV positive vs negative (aHR 1.65 (95%CI 1.55‒1.77) and HIV unknown vs negative (aHR 1.69 (95%CI 1.36‒2.10)).

In the sensitivity analyses, we found evidence of effect modification by HIV status on the association between completing TB treatment outcome and age (*P* < 0.001), TB type (*P* = 0.003) and method of TB diagnosis (*P* = 0.004) Supplementary Table 6. The stratified analyses shown in Supplementary Table 7, highlights some high-risk sub-groups like elderly patients regardless of their HIV status (those aged ≥ 51 years), the extra-pulmonary TB cases and the clinically diagnosed TB patients.

We found evidence of effect modification by HIV status on the association between death and sex (*P* = 0.03), age (*P* < 0.001), TB type (*P* < 0.001), BMI levels (*P* = 0.0001) and method of TB diagnosis (*P* < 0.001) Supplementary Table 6. Supplementary Table 8 highlights the high-risk sub-groups in the stratified analyses (the elderly aged ≥ 51 years either HIV infected or unknown status).

We found evidence of effect modification by HIV status on the association between defaulting TB treatment/LTFU and sex (*P* = 0.02), age (*P* = 0.001) and patient type (*P* = 0.02) Supplementary Table 6. In the stratified analyses, the effects of HIV status on defaulting TB treatment/LTFU varied across many groups as shown on Supplementary Table 9.

## Discussion

The finding of 29% overall burden of TB/HIV co-infection in the two counties is largely comparable with previously pooled estimates across countries in Sub-Saharan Africa [10]. The prevalence was 31% in Tanzania [34], 27.7%, in Amhara region, 37.4% in Addis Ababa and 36.9% in Southern Ethiopia respectively [35], while in Uganda it was 49.2% [36]. The variation in these estimates across the studies could possibly be explained by the differences in study time, sample size, study settings, methods of diagnosis and the ability to systematically screen HIV on all TB patients. Surprisingly despite the different settings of the two counties, the burden was relatively similar.

The TB/HIV co-infection declined during the study period which is in line with findings from other studies [37, 38]. Although many factors can be attributed to this declining trend, the change of policy in testing and treating HIV could be a major factor. Treating all HIV positive cases regardless of their CD4 status reduces the risk of opportunistic infections including TB. Globally and at national level, HIV prevention and control have been strengthened including systematic screening, simplified and readily available diagnosis methods, availability of ARTs and CPT for free to those infected as is reflected in the high uptake in this study and active follow-ups through Mhealth facilities like text messages reminders [39]. Since rolling out the IPT in 2015 in Kenya, the uptake has increased to approximately two-thirds of all PLHIV and scaled up nationally [23]. IPT protects against TB among PLHIV [22], with the increased uptake, it is likely the incidence of TB among PLHIV would decline with time as observed in this study. In the multivariable models, year of starting TB treatment was included as confounder to control for the time effect. Another factor that could have caused the decline in TB/HIV proportion is the overall decrease in TB case notification observed during the study period, for example it dropped by 18% from 7.1million in 2019 to 5.8million in 2020 globally [3]. The drop in TB case notification was more in the year 2020 compared to the previous years. Kenya is one of the 16 countries in the world that contributed largest drop in Global TB case notification in 2020 which was attributed to the impact of COVID-19 pandemic on TB care in the year 2020 [3].

Despite the high uptake of ART and CPT (95% and 99% respectively) in this study, the TB/HIV co-infected patients had poor TB treatment outcomes (80% treatment completion, 12% death and 4.2% lost-to-follow-up) compared to HIV negative patients (86% treatment completion, 5.6% death and 4.4 lost-to-follow-up). The relatively low TB treatment completion among TB/HIV co-infected patients could be due to factors such as HIV treatment failure and poor adherence to both TB and HIV treatment due to high pill burden and drug toxicity or adverse reactions. Studies done in Uganda and Portugal have shown high rates of non-adherence among TB/HIV co-infected patients and that adherence was associated with TB treatment outcome [40, 41]. The occurrence of adverse drug reactions is more frequent in TB/HIV patients concurrently on TB and HIV treatments [42]. Unfortunately, this being a passive surveillance, data on level of drug adherence including the uptake of IPT were not available. The 2-fold higher hazard of death among TB/HIV patients in this study was similar to studies in India and Ethiopia [43, 44]. Similar higher rates of LTFU among TB/HIV patients have been observed in South African patients 12 months after starting ARTs [45]. The interaction between TB and HIV is synergistic, while TB infection worsens HIV-associated immunodeficiency, HIV infection changes the pathogenesis of TB by increasing risk of developing the disease, reactive latent TB, and is association with high incidence of extra pulmonary and atypical radiographic presentation [46, 47]. The exploratory sub-group analysis shows other high-risk subgroups like HIV co-infected patients with extra pulmonary TB and the elderly. As observed in other studies, the CD4 count, WHO HIV stage and disease severity such as being bedridden or ambulatory functional status during treatment are positively associated with risk of death and defaulting treatment, but these data were not available in this study [48].

Although very few patients (1.4%) had unknown HIV status, they were similarly associated with low TB treatment completion and high risk of death and LTFU/defaulting. Screening for HIV among all TB patient is systematically conducted on or during TB treatment. We suspect these missing HIV status were already HIV infected but declined to be tested or were enrolled during HIV testing kits stock out. These results underscore the importance of systematically screening and documenting all TB patients for HIV for proper clinical management. We could not identify when 45% of the HIV cases were diagnosed because it is likely they were diagnosed before 2016 when the policy of immediate ARTs initiation was introduced. However, it is encouraging to observe one quarter of the HIV cases were diagnosed within two weeks of starting TB treatment (two weeks before or after treatment).

### Strengths and limitations

The main strength of this study was the large number of patients systematically followed-up during six months of TB treatment and their outcomes documented. The study was conducted in different geographical and socio-economical settings improving its external validity. While interpreting the findings of this study it is important to note that there are some limitations. This being a passive surveillance, some important data were not collected like CD4 counts or viral load, type of ARTs, IPT uptake, treatment adherence and TB multidrug resistant.

## Conclusion

The overall burden of TB/HIV coinfection was within previous pooled estimate. HIV infection and unknown HIV status were associated with lower TB treatment completion rates and higher risk of death and defaulting TB treatment despite the high uptake of ARTs and cotrimoxazole preventive therapy. Our findings support the need for systematic HIV testing when starting TB treatment and more studies to investigate interventions targeting TB treatment completion and reducing mortality.

## Supplementary Information

**Additional file 1.**

## Data Availability

The datasets used and/or analysed during the current study are available from the corresponding author on reasonable request.

## Abbreviations

ART: Antiretroviral therapy
BMI: Body Mass Index
CCC: Comprehensive Care Center
CPT: Cotrimoxazole preventive therapy
DR TB: Drug resistant TB
DOT: Directly observed therapy
HIV: Human immunodeficiency virus
HR: Hazard ratio
IPT: Isoniazid preventive therapy
LTFU: Lost to follow-up
MDR/RR: Multidrug-resistant/rifampicin-resistant
OR: Odds ratio
PITC: Provider-initiated testing & counselling
PLHIV: People living with HIV
RDT: Rapid diagnosis test
TB: Tuberculosis
VCT: Voluntary counselling & testing
WHO: World Health Organization
