# Supplementary material for: Burden of HIV and treatment outcomes among TB patients in rural Kenya: a 9-year longitudinal study
Source: BMC Infect Dis. 2023 May 30;23:362. doi: 10.1186/s12879-023-08347-0 (PMC10227789; doi:10.1186/s12879-023-08347-0)
Supplement: Supplementary file 1 — Additional file 1. [file 12879_2023_8347_MOESM1_ESM.docx]

**Supplementary materials**

STROBE Statement—checklist of items that should be included in reports of observational studies

|  | Item No | Recommendation |
| --- | --- | --- |
| **Title and abstract** | 1 | (*a*) Indicate the study’s design with a commonly used term in the title or the abstract (**Page 2, method section of the abstract**) |
|  |  | (*b*) Provide in the abstract an informative and balanced summary of what was done and what was found. (**Page 2, the abstract**) |
| Introduction | | |
| Background/rationale | 2 | Explain the scientific background and rationale for the investigation being reported. (**Pages 4 and 5 of the background section**) |
| Objectives | 3 | State specific objectives, including any prespecified hypotheses (**Page 5, the last paragraph of background section**) |
| Methods | | |
| Study design | 4 | Present key elements of study design early in the paper (**Page 6, the first paragraph of methods section**) |
| Setting | 5 | Describe the setting, locations, and relevant dates, including periods of recruitment, exposure, follow-up, and data collection (**Page 6, study settings sub-section)** |
| Participants | 6 | (*a*) *Cohort study*—Give the eligibility criteria, and the sources and methods of selection of participants. Describe methods of follow-up. (**Page 7 under study population sub-section**)  *Case-control study*—Give the eligibility criteria, and the sources and methods of case ascertainment and control selection. Give the rationale for the choice of cases and controls  *Cross-sectional study*—Give the eligibility criteria, and the sources and methods of selection of participants |
|  |  | (*b*) *Cohort study*—For matched studies, give matching criteria and number of exposed and unexposed. (**Page 7, data source and variables sub-section**)  *Case-control study*—For matched studies, give matching criteria and the number of controls per case |
| Variables | 7 | Clearly define all outcomes, exposures, predictors, potential confounders, and effect modifiers. Give diagnostic criteria, if applicable. (**Page 7, data source and variables sub-section and pages 8 & 9, statistical methods**) |
| Data sources/ measurement | 8* | For each variable of interest, give sources of data and details of methods of assessment (measurement). Describe comparability of assessment methods if there is more than one group. (**Page 7, data source and variables sub-section**) |
| Bias | 9 | Describe any efforts to address potential sources of bias (P**ages 8 & 9, statistical methods)** |
| Study size | 10 | Explain how the study size was arrived at. (**Page 8, study size sub-section**) |
| Quantitative variables | 11 | Explain how quantitative variables were handled in the analyses. If applicable, describe which groupings were chosen and why. (**Page 7, data source and variables sub-section and pages 8 & 9, statistical methods**) |
| Statistical methods | 12 | (*a*) Describe all statistical methods, including those used to control for confounding (P**ages 8 & 9, statistical methods)** |
|  |  | (*b*) Describe any methods used to examine subgroups and interactions (P**age 9, statistical methods)** |
|  |  | (*c*) Explain how missing data were addressed (P**age 8, statistical methods)** |
|  |  | (*d*) *Cohort study*—If applicable, explain how loss to follow-up was addressed (P**ages 8 & 9, statistical methods)**  *Case-control study*—If applicable, explain how matching of cases and controls was addressed  *Cross-sectional study*—If applicable, describe analytical methods taking account of sampling strategy |
|  |  | (*e*) Describe any sensitivity analyses (P**age 9, statistical methods)** |

Continued on next page

| Results | | |
| --- | --- | --- |
| Participants | 13* | (a) Report numbers of individuals at each stage of study—eg numbers potentially eligible, examined for eligibility, confirmed eligible, included in the study, completing follow-up, and analysed (**Page 10, Results section**) |
|  |  | (b) Give reasons for non-participation at each stage - |
|  |  | (c) Consider use of a flow diagram |
| Descriptive data | 14* | (a) Give characteristics of study participants (eg demographic, clinical, social) and information on exposures and potential confounders (**Page 10, Table 1**) |
|  |  | (b) Indicate number of participants with missing data for each variable of interest (**Page 10, Table 1**) |
|  |  | (c) *Cohort study*—Summarise follow-up time (eg, average and total amount) (**Page 11, paragraph 3)** |
| Outcome data | 15* | *Cohort study*—Report numbers of outcome events or summary measures over time (**Page 10, results section)** |
|  |  | *Case-control study—*Report numbers in each exposure category, or summary measures of exposure |
|  |  | *Cross-sectional study—*Report numbers of outcome events or summary measures |
| Main results | 16 | (*a*) Give unadjusted estimates and, if applicable, confounder-adjusted estimates and their precision (eg, 95% confidence interval). Make clear which confounders were adjusted for and why they were included (**Pages 10 to 11, results section**) |
|  |  | (*b*) Report category boundaries when continuous variables were categorized |
|  |  | (*c*) If relevant, consider translating estimates of relative risk into absolute risk for a meaningful time period **N/A** |
| Other analyses | 17 | Report other analyses done—eg analyses of subgroups and interactions, and sensitivity analyses (**Pages 12, results section**) |
| Discussion | | |
| Key results | 18 | Summarise key results with reference to study objectives (**Pages 13 & 14 of discussion section)** |
| Limitations | 19 | Discuss limitations of the study, taking into account sources of potential bias or imprecision. Discuss both direction and magnitude of any potential bias (**Pages 14 & 15**) |
| Interpretation | 20 | Give a cautious overall interpretation of results considering objectives, limitations, multiplicity of analyses, results from similar studies, and other relevant evidence (**Pages 13 to 14)** |
| Generalisability | 21 | Discuss the generalisability (external validity) of the study results (**Pages 14 & 15**) |
| Other information | | |
| Funding | 22 | Give the source of funding and the role of the funders for the present study and, if applicable, for the original study on which the present article is based |

*Give information separately for cases and controls in case-control studies and, if applicable, for exposed and unexposed groups in cohort and cross-sectional studies.

**Note:** An Explanation and Elaboration article discusses each checklist item and gives methodological background and published examples of transparent reporting. The STROBE checklist is best used in conjunction with this article (freely available on the Web sites of PLoS Medicine at http://www.plosmedicine.org/, Annals of Internal Medicine at http://www.annals.org/, and Epidemiology at http://www.epidem.com/). Information on the STROBE Initiative is available at www.strobe-statement.org.

Supplementary Table 1. **Test of Proportion hazard assumption.**

| **TB treatment outcome** | **PH test P-value in the univariate model** | **Global PH test P-value in the multivariable model** |
| --- | --- | --- |
| Treatment failure | 0.98 | 0.38 |
| Died | 0.19 | 0.09 |
| Default | 0.87 | 0.12 |
| Transfer out | 0.09 | 0.43 |
| Proportion hazard (PH) assumption P-value from the schoenfeld residuals test, multivariable models were adjusted for age, sex, patient type, TB type, facility type, method of DOT, BMI, TB treatment regimen, type of TB diagnosis, presence of other underlying medical conditions and year of diagnosis. | | |

Supplementary Table 2: **Multivariable analysis of the effect of HIV status on TB treatment completion rates.**

| **Variable** | **Adjusted Odds Ratio** | **95% CI** | **P-value** |
| --- | --- | --- | --- |
| HIV status |  |  |  |
| Negative | Reference |  |  |
| Positive | 0.56 | 0.52‒0.61 | <0.001 |
| Unknown | 0.57 | 0.44‒0.73 | <0.001 |
| Sex |  |  |  |
| Male | Reference |  |  |
| Female | 1.17 | 1.08‒1.26 | <0.001 |
| Age in years |  |  |  |
| 18 to 30 | Reference |  |  |
| 31 to 40 | 1.04 | 0.95‒1.14 | 0.44 |
| 41 to 50 | 0.97 | 0.87‒1.07 | 0.53 |
| >50 | 0.64 | 0.59‒0.71 | <0.001 |
| Patient type |  |  |  |
| New case | Reference |  |  |
| Existing case | 0.87 | 0.76‒0.98 | 0.03 |
| TB type |  |  |  |
| Pulmonary TB | Reference |  |  |
| Extrapulmonary TB | 0.98 | 0.88‒1.08 | 0.68 |
| Facility type |  |  |  |
| Public | Reference |  |  |
| Private | 1.01 | 0.93‒1.10 | 0.77 |
| Prisons | 1.56 | 1.15‒2.11 | 0.004 |
| DOT type |  |  |  |
| Family-based | Reference |  |  |
| Community volunteer | 0.91 | 0.75‒1.11 | 0.38 |
| Health worker | 0.92 | 0.77‒1.11 | 0.38 |
| BMI group |  |  |  |
| Undernourished | 0.94 | 0.87‒1.01 | 0.10 |
| Normal BMI | Reference |  |  |
| Overweight | 0.99 | 0.88‒1.11 | 0.84 |
| Not reported | 0.48 | 0.42‒0.54 | <0.001 |
| Treatment regimen |  |  |  |
| 2RHZE/4RH | Reference |  |  |
| 2SRHZE/1RHZE/5RHE | 0.81 | 0.67‒0.98 | 0.03 |
| 2RHZ/4RH | 1.00 | 0.77‒1.30 | 0.98 |
| Others | 0.92 | 0.62‒1.36 | 0.67 |
| Method of TB diagnosis |  |  |  |
| Bacteriological confirmed | Reference |  |  |
| Clinical signs | 0.79 | 0.73‒0.85 | <0.001 |
| Underlying medical condition |  |  |  |
| No | Reference |  |  |
| Yes | 0.52 | 0.42‒0.65 | <0.001 |
| Year of diagnosis |  |  |  |
| 2012 | Reference |  |  |
| 2013 | 1.01 | 0.86‒1.20 | 0.87 |
| 2014 | 0.67 | 0.57‒0.77 | <0.001 |
| 2015 | 0.58 | 0.50‒0.68 | <0.001 |
| 2016 | 0.41 | 0.35‒0.48 | <0.001 |
| 2017 | 0.39 | 0.34‒0.45 | <0.001 |
| 2018 | 0.36 | 0.31‒0.41 | <0.001 |
| 2019 | 0.42 | 0.36‒0.49 | <0.001 |
| 2020 | 0.58 | 0.48‒0.68 | <0.001 |

Supplementary Table 3: **Multivariable analysis of the effect of HIV status on all-cause deaths.**

| **Variable** | **Adjusted Hazard Ratio** | **95% CI** | **P-value** |
| --- | --- | --- | --- |
| HIV status |  |  |  |
| Negative | Reference |  |  |
| Positive | 2.40 | 2.18‒2.63 | <0.001 |
| Unknown | 1.93 | 1.44‒2.58 | <0.001 |
| Sex |  |  |  |
| Male | Reference |  |  |
| Female | 0.91 | 0.82‒0.99 | 0.04 |
| Age in years |  |  |  |
| 18 to 30 | Reference |  |  |
| 31 to 40 | 1.33 | 1.15‒1.54 | <0.001 |
| 41 to 50 | 1.76 | 1.52‒2.04 | <0.001 |
| >50 | 3.40 | 2.98‒3.88 | <0.001 |
| Patient type |  |  |  |
| New case | Reference |  |  |
| Existing case | 1.01 | 0.86‒1.19 | 0.89 |
| TB type |  |  |  |
| Pulmonary TB | Reference |  |  |
| Extrapulmonary TB | 1.06 | 0.95‒1.19 | 0.29 |
| Facility type |  |  |  |
| Public | Reference |  |  |
| Private | 1.03 | 0.93‒1.14 | 0.61 |
| Prisons | 0.32 | 0.18‒0.57 | <0.001 |
| DOT type |  |  |  |
| Family-based | Reference |  |  |
| Community volunteer | 0.83 | 0.60‒1.14 | 0.25 |
| Health worker | 1.21 | 0.95‒1.53 | 0.12 |
| BMI group |  |  |  |
| Undernourished | 0.95 | 0.86‒1.06 | 0.40 |
| Normal BMI | Reference |  |  |
| Overweight | 1.01 | 0.88‒1.16 | 0.87 |
| Not reported | 2.11 | 1.83‒2.42 | <0.001 |
| Treatment regimen |  |  |  |
| 2RHZE/4RH | Reference |  |  |
| 2SRHZE/1RHZE/5RHE | 1.17 | 0.92‒1.47 | 0.19 |
| 2RHZ/4RH | 0.98 | 0.69‒1.39 | 0.92 |
| Others | 1.10 | 0.74‒1.65 | 0.64 |
| Method of TB diagnosis |  |  |  |
| Bacteriological confirmed | Reference |  |  |
| Clinical signs | 2.20 | 1.98‒2.44 | <0.001 |
| Underlying medical condition |  |  |  |
| No | Reference |  |  |
| Yes | 2.03 | 1.61‒2.56 | <0.001 |
| Year of diagnosis |  |  |  |
| 2012 | Reference |  |  |
| 2013 | 1.10 | 0.91‒1.34 | 0.34 |
| 2014 | 1.21 | 1.00‒1.47 | 0.05 |
| 2015 | 1.27 | 1.04‒1.55 | 0.02 |
| 2016 | 1.67 | 1.36‒2.04 | <0.001 |
| 2017 | 1.89 | 1.57‒2.29 | <0.001 |
| 2018 | 1.88 | 1.57‒2.25 | <0.001 |
| 2019 | 1.85 | 1.54‒2.23 | <0.001 |
| 2020 | 1.38 | 1.12‒1.70 | 0.002 |

Supplementary Table 4: **Multivariable analysis of the effect of HIV status on defaulting/Lost to follow-up.**

| **Variable** | **Adjusted Hazard Ratio** | **95% CI** | **P-value** |
| --- | --- | --- | --- |
| HIV status |  |  |  |
| Negative | Reference |  |  |
| Positive | 1.16 | 1.01‒1.32 | 0.04 |
| Unknown | 1.55 | 1.02‒2.35 | 0.04 |
| Sex |  |  |  |
| Male | Reference |  |  |
| Female | 0.72 | 0.63‒0.83 | <0.001 |
| Age in years |  |  |  |
| 18 to 30 | Reference |  |  |
| 31 to 40 | 0.90 | 0.78‒1.04 | 0.16 |
| 41 to 50 | 0.68 | 0.57‒0.81 | <0.001 |
| >50 | 0.66 | 0.56‒0.79 | <0.001 |
| Patient type |  |  |  |
| New case | Reference |  |  |
| Existing case | 1.79 | 1.49‒2.16 | <0.001 |
| TB type |  |  |  |
| Pulmonary TB | Reference |  |  |
| Extrapulmonary TB | 0.94 | 0.77‒1.16 | 0.59 |
| Facility type |  |  |  |
| Public | Reference |  |  |
| Private | 0.93 | 0.80‒1.09 | 0.38 |
| Prisons | 0.69 | 0.42‒1.15 | 0.15 |
| DOT type |  |  |  |
| Family-based | Reference |  |  |
| Community volunteer | 1.23 | 0.93‒1.62 | 0.15 |
| Health worker | 1.04 | 0.75‒1.43 | 0.83 |
| BMI group |  |  |  |
| Undernourished | 1.11 | 0.97‒1.26 | 0.12 |
| Normal BMI | Reference |  |  |
| Overweight | 0.92 | 0.74‒1.14 | 0.46 |
| Not reported | 1.60 | 1.29‒1.99 | <0.001 |
| Treatment regimen |  |  |  |
| 2RHZE/4RH | Reference |  |  |
| 2SRHZE/1RHZE/5RHE | 0.54 | 0.39‒0.74 | <0.001 |
| 2RHZ/4RH | 1.04 | 0.68‒1.57 | 0.87 |
| Others | 0.91 | 0.40‒2.06 | 0.82 |
| Method of TB diagnosis |  |  |  |
| Bacteriological confirmed | Reference |  |  |
| Clinical signs | 0.87 | 0.77‒1.00 | 0.05 |
| Underlying medical condition |  |  |  |
| No | Reference |  |  |
| Yes | 2.29 | 1.66‒3.15 | <0.001 |
| Year of diagnosis |  |  |  |
| 2012 | Reference |  |  |
| 2013 | 0.16 | 0.09‒1.89 | 0.97 |
| 2014 | 0.38 | 0.29‒0.52 | <0.001 |
| 2015 | 0.50 | 0.37‒0.67 | <0.001 |
| 2016 | 0.68 | 0.51‒0.90 | <0.001 |
| 2017 | 0.73 | 0.56‒0.96 | <0.001 |
| 2018 | 0.13 | 0.09‒0.16 | <0.001 |
| 2019 | 0.86 | 0.66‒1.12 | 0.47 |
| 2020 | 0.39 | 0.07‒1.12 | 0.52 |

Supplementary Table 5: **Univariate and multivariable analysis of the effect of HIV status on TB treatment outcomes among HIV patients on ARTs and Cotrimoxazole prophylaxis.**

| **TB treatment outcome** | **Univariate analysis** | | **Multivariable analysis** | |
| --- | --- | --- | --- | --- |
|  |  | **P-value** |  | **P-value** |
|  | **Crude Odds Ratio**  **(95% CI)** |  | **Adjusted Odds ratio**  **(95% CI)#** |  |
| **Treatment complete** |  |  |  |  |
| HIV positive Vs negative | 0.65 (0.60‒0.67) | <0.001 | 0.59 (0.55‒0.64) | <0.001 |
| HIV unknown Vs negative | 0.56 (0.44‒0.71) | <0.001 | 0.57 (0.44‒0.73) | <0.001 |
|  | **Crude HR**  **(95% CI)** |  | **Adjusted HR (95% CI)#** |  |
| **Died** |  |  |  |  |
| HIV positive Vs negative | 2.21 (2.02‒2.42) | <0.001 | 2.27 (2.06‒2.51) | <0.001 |
| HIV unknown Vs negative | 2.34 (1.75‒3.13) | <0.001 | 1.93 (1.44‒2.59) | <0.001 |
| **Defaulted/Lost-to-follow-up** |  |  |  |  |
| HIV positive Vs negative | 0.99 (0.87‒1.13) | 0.90 | 1.15 (1.00‒1.32) | 0.05 |
| HIV unknown Vs negative | 1.58 (1.04‒2.39) | 0.03 | 1.55 (1.02‒2.35) | 0.04 |
| **Transfer out** |  |  |  |  |
| HIV positive Vs negative | 1.02 (0.87‒1.20) | 0.82 | 1.05 (0.89‒1.25) | 0.56 |
| HIV unknown Vs negative | 1.08 (0.59‒1.96) | 0.80 | 1.01 (0.55‒1.83) | 0.98 |
| **Treatment failure** |  |  |  |  |
| HIV positive Vs negative | 0.87 (0.65‒1.16) | 0.33 | 1.10 (0.81‒1.50) | 0.52 |
| HIV unknown Vs negative | 1.18 (0.44‒3.19) | 0.74 | 1.53 (0.57‒4.18) | 0.39 |
| #Adjusted for *a priori* confounders: age, sex, patient type, TB type, facility type, method of DOT, BMI, TB treatment regimen, type of TB diagnosis, presence of other underlying medical conditions and year of diagnosis, Odds Ratios are from multilevel logit regression model, Hazard Ratios are from Cox proportion regression models. | | | | |

Supplementary Table 6. **Test of effect modification with HIV status.**

| **Exposure variables** | **TB treatment outcome test of effect modification P-value** | | |
| --- | --- | --- | --- |
|  | Treatment complete | Died | Default/lost-to-follow-up |
| Sex | 0.11 | 0.03 | 0.02 |
| Age in years | <0.001 | <0.001 | 0.001 |
| Patient type | 0.97 | 0.52 | 0.02 |
| TB type | 0.003 | <0.001 | 0.96 |
| BMI | 0.50 | 0.0001 | 0.11 |
| Treatment regimen | 0.09 | 0.98 | 0.10 |
| Method of TB diagnosis | 0.0004 | <0.001 | 0.47 |
| Underlying medical conditions | 0.99 | 0.26 | 0.90 |
| Effect modification was conducted by comparing model with and without interaction term using likelihood-ratio test, the effect modification was conducted on selected variables and on only two TB treatment outcomes that were significantly associated with HIV status. | | | |

Supplementary Table 7. **The effect modification of HIV status with selected exposure variables on TB treatment complete.**

| **Interaction terms** | **Effect on TB treatment complete outcome; adjusted Odds ratios (95% CI)** | **P-value** |
| --- | --- | --- |
| Age in years |  |  |
| HIV negative + age ≤30 years | Reference |  |
| HIV negative + age 31 to 40 years | 1.01 (0.90‒1.14) | 0.84 |
| HIV negative + age 41 to 50 years | 0.86 (0.75‒0.98) | 0.03 |
| HIV negative + age ≥51 years | 0.52 (0.47‒0.58) | <0.001 |
| HIV positive + age ≤30 years | 0.40 (0.35‒0.46) | <0.001 |
| HIV positive + age 31 to 40 years | 0.51 (0.45‒0.57) | <0.001 |
| HIV positive + age 41 to 50 years | 0.50 (0.43‒0.57) | <0.001 |
| HIV positive + age ≥51 years | 0.44 (0.37‒0.52) | <0.001 |
| HIV unknown + age ≤30 years | 1.55 (0.78‒3.09) | 0.21 |
| HIV unknown + age 31 to 40 years | 0.29 (0.18‒0.45) | <0.001 |
| HIV unknown + age 41 to 50 years | 0.59 (0.30‒1.15) | 0.12 |
| HIV unknown + age ≥51 years | 0.27 (0.17‒0.40) | <0.001 |
| TB type |  |  |
| HIV negative + Pulmonary TB | Reference |  |
| HIV negative + Extra-pulmonary TB | 0.79 (0.70‒0.89) | <0.001 |
| HIV positive + Pulmonary TB | 0.53 (0.49‒0.57) | <0.001 |
| HIV positive + Extra-pulmonary TB | 0.55 (0.47‒0.64) | <0.001 |
| HIV unknown + Pulmonary TB | 0.58 (0.43‒0.76) | <0.001 |
| HIV unknown + Extra-pulmonary TB | 0.43 (0.24‒0.75) | 0.003 |
| Method of TB diagnosis |  |  |
| HIV negative + Bacteriological confirmed | Reference |  |
| HIV negative + Clinical signs | 0.75 (0.68‒0.82) | <0.001 |
| HIV positive + Bacteriological confirmed | 0.52 (0.47‒0.58) | <0.001 |
| HIV positive + Clinical signs | 0.45 (0.41‒0.50) | <0.001 |
| HIV Unknown + Bacteriological confirmed | 0.63 (0.42‒0.93) | 0.02 |
| HIV Unknown + Clinical signs | 0.40 (0.29‒0.56) | <0.001 |

Supplementary Table 8. **The effect modification of HIV status with selected exposure variables on deaths.**

| **Interaction terms** | **Effect on death; adjusted Hazard ratios (95% CI)** | **P-value** |
| --- | --- | --- |
| Sex |  |  |
| HIV negative + male | Reference |  |
| HIV negative + female | 0.90 (0.79‒1.03) | 0.14 |
| HIV positive + male | 2.41 (2.15‒2.71) | <0.001 |
| HIV positive + female | 2.15 (1.90‒2.43) | <0.001 |
| HIV unknown + male | 1.65 (1.13‒2.41) | 0.01 |
| HIV unknow + female | 2.28 (1.45‒3.57) | <0.001 |
| Age in years |  |  |
| HIV negative + age ≤30 years | Reference |  |
| HIV negative + age 31 to 40 years | 1.70 (1.33‒2.16) | <0.001 |
| HIV negative + age 41 to 50 years | 2.62 (2.05‒3.34) | <0.001 |
| HIV negative + age ≥51 years | 6.34 (5.20‒7.72) | <0.001 |
| HIV positive + age ≤30 years | 6.15 (4.89‒7.76) | <0.001 |
| HIV positive + age 31 to 40 years | 5.44 (4.40‒6.73) | <0.001 |
| HIV positive + age 41 to 50 years | 6.74 (5.44‒8.34) | <0.001 |
| HIV positive + age ≥51 years | 8.15 (6.46‒10.3) | <0.001 |
| HIV unknown + age ≤30 years | 0.42 (0.06‒2.99) | 0.39 |
| HIV unknown + age 31 to 40 years | 5.52 (2.89‒10.5) | <0.001 |
| HIV unknown + age 41 to 50 years | 4.81 (2.12‒10.9) | <0.001 |
| HIV unknown + age ≥51 years | 11.9 (7.96‒17.7) | <0.001 |
| Patient type |  |  |
| HIV negative + new cases | Reference |  |
| HIV negative + re-treatment cases | 0.95 (0.77‒1.17) | 0.64 |
| HIV positive + new cases | 2.35 (2.13‒2.60) | <0.001 |
| HIV positive + re-treatment cases | 2.54 (2.07‒3.12) | <0.001 |
| HIV unknown + new cases | 1.92 (1.42‒2.61) | <0.001 |
| HIV unknown + re-treatment cases | 1.82 (0.68‒4.90) | 0.23 |
| TB type |  |  |
| HIV negative + Pulmonary TB | Reference |  |
| HIV negative + Extra-pulmonary TB | 1.32 (1.14‒1.54) | <0.001 |
| HIV positive + Pulmonary TB | 2.64 (2.38‒2.93) | <0.001 |
| HIV positive + Extra-pulmonary TB | 2.11 (1.76‒2.54) | <0.001 |
| HIV unknown + Pulmonary TB | 1.87 (1.32‒2.65) | <0.001 |
| HIV unknown + Extra-pulmonary TB | 2.63 (1.57‒4.43) | <0.001 |
| BMI |  |  |
| HIV negative + Normal BMI | Reference |  |
| HIV negative + Undernourished | 0.90 (0.77‒1.05) | 0.17 |
| HIV negative + Overweight | 1.17 (0.98‒1.40) | 0.09 |
| HIV negative + Not reported | 2.08 (1.71‒2.53) | <0.001 |
| HIV positive + Normal BMI | 2.41 (2.12‒2.75) | <0.001 |
| HIV positive + Undernourished | 2.43 (2.09‒2.83) | <0.001 |
| HIV positive + Overweight | 2.00 (1.60‒2.52) | <0.001 |
| HIV positive + Not reported | 5.21 (4.23‒6.41) | <0.001 |
| HIV unknown + Normal BMI | 1.97 (1.26‒3.09) | 0.003 |
| HIV unknown + Undernourished | 2.04 (1.05‒3.95) | 0.03 |
| HIV unknown + Overweight | 1.68 (0.63‒4.51) | 0.30 |
| HIV unknown + Not reported | 3.93 (2.35‒6.58) | <0.001 |
| Method of TB diagnosis |  |  |
| HIV negative + Bacteriological confirmed | Reference |  |
| HIV negative + Clinical signs | 3.07 (2.66‒3.55) | <0.001 |
| HIV positive + Bacteriological confirmed | 3.91 (3.31‒4.62) | <0.001 |
| HIV positive + Clinical signs | 5.99 (5.16‒6.96) | <0.001 |
| HIV Unknown + Bacteriological confirmed | 2.06 (1.10‒3.89) | 0.03 |
| HIV Unknown + Clinical signs | 5.65 (4.00‒7.99) | <0.001 |

Supplementary Table 9. **The effect modification of HIV status with selected exposure variables on defaulting TB treatment/lost-to-follow-up.**

| **Interaction terms** | **Effect on death; adjusted Hazard ratios (95% CI)** | **P-value** |
| --- | --- | --- |
| Sex |  |  |
| HIV negative + male | Reference |  |
| HIV negative + female | 0.65 (0.54‒0.77) | <0.001 |
| HIV positive + male | 1.05 (0.88‒1.25) | 0.60 |
| HIV positive + female | 0.95 (0.79‒1.13) | 0.55 |
| HIV unknown + male | 1.83 (1.13‒2.97) | 0.02 |
| HIV unknow + female | 1.60 (0.71‒3.59) | 0.25 |
| Age in years |  |  |
| HIV negative + age ≤30 years | Reference |  |
| HIV negative + age 31 to 40 years | 0.83 (0.70‒0.99) | 0.04 |
| HIV negative + age 41 to 50 years | 0.74 (0.60‒0.92) | 0.006 |
| HIV negative + age ≥51 years | 0.68 (0.56‒0.82) | <0.001 |
| HIV positive + age ≤30 years | 1.51 (1.20‒1.89) | <0.001 |
| HIV positive + age 31 to 40 years | 1.16 (0.95‒1.41) | 0.14 |
| HIV positive + age 41 to 50 years | 0.66 (0.50‒0.86) | 0.003 |
| HIV positive + age ≥51 years | 0.41 (0.27‒0.64) | <0.001 |
| HIV unknown + age ≤30 years | 0.67 (0.22‒2.10) | 0.50 |
| HIV unknown + age 31 to 40 years | 3.70 (2.11‒6.47) | <0.001 |
| HIV unknown + age 41 to 50 years | 0.93 (0.23‒3.75) | 0.92 |
| HIV unknown + age ≥51 years | 1.23 (0.50‒2.98) | 0.65 |
| Patient type |  |  |
| HIV negative + new cases | Reference |  |
| HIV negative + re-treatment cases | 1.37 (1.11‒1.69) | 0.004 |
| HIV positive + new cases | 1.29 (1.11‒1.49) | 0.001 |
| HIV positive + re-treatment cases | 1.08 (0.77‒1.50) | 0.67 |
| HIV unknown + new cases | 2.11 (1.37‒3.23) | 0.001 |
| HIV unknown + re-treatment cases | 1.22 (0.17‒8.72) | 0.85 |
